# Nomogram to predict multidrug-resistant tuberculosis

**DOI:** 10.1186/s12941-020-00369-9

**Published:** 2020-06-06

**Authors:** Saibin Wang, Junwei Tu

**Affiliations:** Department of Respiratory Medicine, Jinhua Municipal Central Hospital, Jinhua Hospital of Zhejiang University, No. 365, East Renmin Road, Jinhua, 321000 Zhejiang Province China

**Keywords:** MDR-TB, Prediction, Infection

## Abstract

**Background:**

Multidrug-resistant tuberculosis (MDR-TB) is burgeoning globally, and has been a serious challenge in TB management. Clinically, the ability to identify MDR-TB is still limited, especially in smear-negative TB. The aim of this study was to develop a nomogram for predicting MDR-TB.

**Methods:**

Demographics and clinical characteristics of both MDR-TB and drug-susceptible TB patients were utilized to develop a nomogram for predicting MDR-TB. The LASSO regression method was applied to filter variables and select predictors, and multivariate logistic regression was used to construct a nomogram. The discriminatory ability of the model was determined by calculating the area under the curve (AUC). Moreover, calibration analysis and decision curve analysis (DCA) of the model were performed. This study involved a second analysis of a completed prospective cohort study conducted in a country with a high TB burden.

**Results:**

Five variables of TB patients were selected through the LASSO regression method, and a nomogram was built based on these variables. The predictive model yielded an AUC of 0.759 (95% CI, 0.719–0.799), and in the internal validation, the AUC was 0.757 (95% CI, 0.715–0.793). The predictive model was well-calibrated, and DCA showed that if the threshold probability of MDR-TB was between 70 and 90%, using the proposed nomogram to predict MDR-TB would obtain a net benefit.

**Conclusions:**

In this study, a nomogram was constructed that incorporated five demographic and clinical characteristics of TB patients. The nomogram may be of great value for the prediction of MDR-TB in patients with sputum-free or smear-negative TB.

## Background

Tuberculosis (TB) continues to be a heavy burden globally, and alarmingly, the epidemic of resistance is burgeoning [1]. Multidrug-resistant TB (MDR-TB) was defined as resistance to at least isoniazid and rifampin. There were approximately 458,000 new prevalent cases of multidrug-resistant TB (MDR-TB) globally in 2017 [1]. The mortality of MDR-TB in endemic countries or regions using traditional regimens reached 40% [2]. It is much costlier to treat MDR-TB than drug-susceptible TB (DS-TB) [1, 3]. There is a difference in regimen and management between MDR-TB and DS-TB, and therefore it is crucial to identify MDR-TB. Although the diagnostic capability of MDR-TB increased by the phenotypic drug susceptibility testing (DST), array-based platforms and line probe assays [4, 5], missed diagnosis or a delayed diagnosis of MDR-TB is still common in clinical practice, especially in sputum-free or smear-negative TB [6].

In general, MDR-TB is mainly acquired (caused by improper treatment programs or poor patient treatment compliance); however, primary transmission of MDR-TB could be a dominant mode of spread in epidemics [7, 8]. It has been revealed that several clinical, environmental, and socioeconomic characteristics were different between cases with MDR-TB and DS-TB [9]. In addition, it has previously been pointed out that the pathogenicity of DR and DS *Mycobacterium tuberculosis* differs in both animal models and human patients [9–11]. To accurately identify MDR-TB and start an appropriate treatment regimen is essential in MDR-TB control. However, to the best of our knowledge, there is currently no model available for the prediction of MDR-TB.

In the present study, we developed a nomogram for predicting MDR-TB based on demographic and clinical characteristics of TB patients, which were collected from a completed 3-year prospective cohort study.

## Materials and methods

### Study population and ethics

This was an analytical study of a previously completed prospective cohort study that was conducted in Peru, which is a country with a high TB burden as defined by the WHO [1]. In the present study, a total of 700 confirmed TB patients were enrolled between September 2010 and September 2013 in Peru [9]. MDR-TB referred to resistance to at least rifampicin and isoniazid, whereas DS-TB referred to susceptibility to both rifampicin and isoniazid [9]. Patients who expectorated sputum had their sputum tested by DST for rifampicin and isoniazid using the microscopic observation drug susceptibility assay (MODS) [12, 13], in which MODS testing indicated resistance to rifampicin and isoniazid would be confirmed by a second test. All TB patients underwent a human immunodeficiency virus (HIV) test [9]. Ethical approval of this secondary analysis was obtained from the Jinhua Municipal Central Hospital (Jinhua, China). Informed consent was waived because the data used in this study were publicly available from a public database [14], and patient information was anonymous.

The variables of TB patients collected for further analysis were as follows: gender, smoking, alcohol use, coexisting diabetes, HIV infection status, previous TB history, socioeconomic status (divided into three levels based on the scoring system used in the Peruvian National Census) [9], employment status (unemployed, working, student, or unknown), secondary education status (completed or not), history of incarceration, sputum smear grade, mean cough duration, hospitalization history, side effects of treatment (yes/no), and spoligotypes (stratified based on SpolDB4 database) [9].

### Statistical analysis

Continuous data and categorical data were expressed as the median (interquartile) and the number (proportion), respectively. Group comparisons between MDR-TB and DS-TB were performed using an unpaired *t* test or Wilcoxon rank sum test, Pearson Chi square test or the Fisher’s exact test, as appropriate. The least absolute shrinkage and selection operator (LASSO) regression technique was used for data dimension and predictor selection. Multivariable logistic regression analysis was used to develop a predictive model and a nomogram of MDR-TB. The discriminatory capacity of the model was determined by calculating the area under the curve (AUC). The bootstrapping method (resampling = 500) was employed for internal validation [15]. The calibration of the model was evaluated by using the Hosmer–Lemeshow test, and the clinical usefulness of the model was assessed by decision curve analysis (DCA) [16]. Statistical analysis was conducted using R software (version 3.5.1; R Foundation for Statistical Computing, Vienna, Austria), and P < 0.05 was considered statistically significant.

## Results

**Fig. 1:**
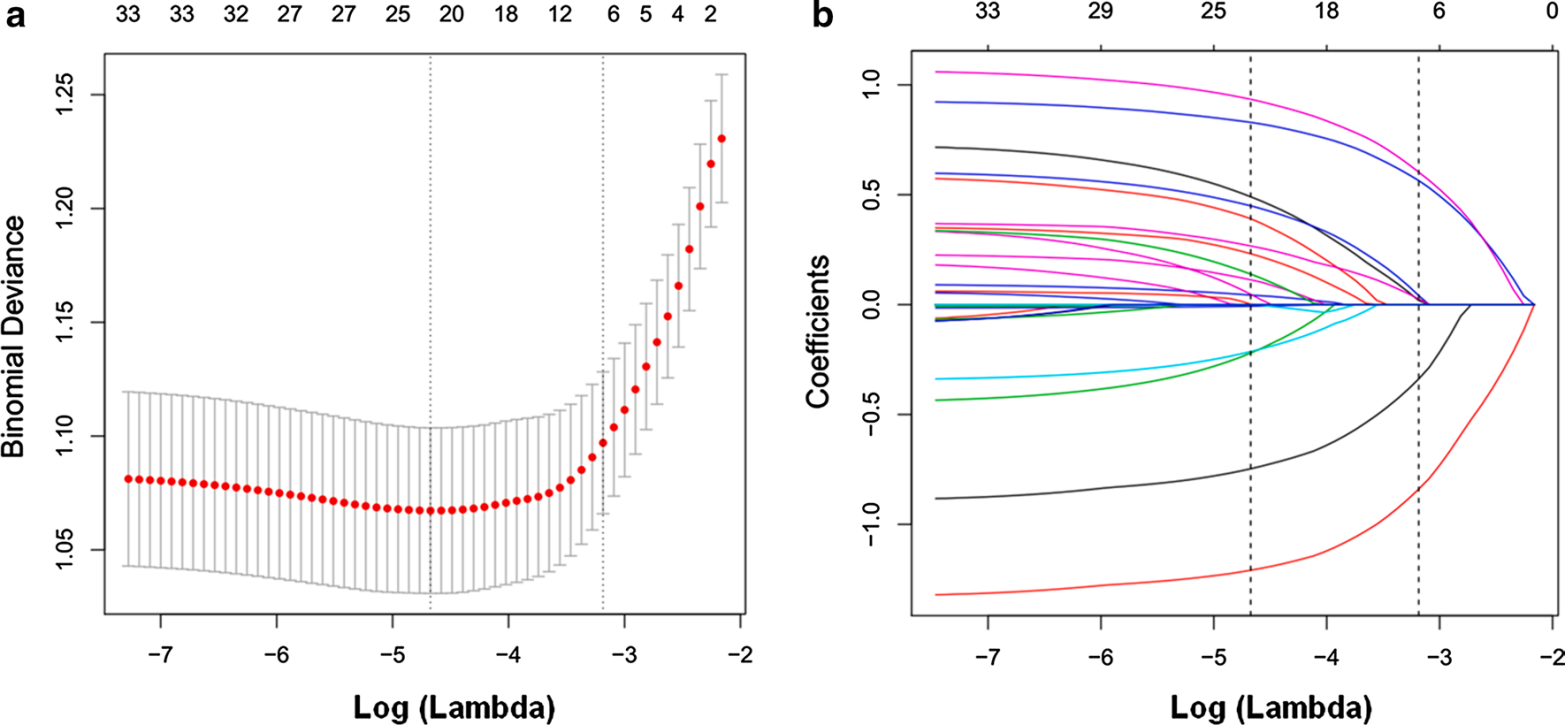
Predictor selection using the LASSO regression analysis with tenfold cross-validation. (A) Tuning parameter (lambda) selection of deviance in the LASSO regression based on the minimum criteria (left dotted line) and the 1-SE criteria (right dotted line). (B) A coefficient profile plot was created against the log (lambda) sequence. In the present study, predictor’s selection was according to the 1-SE criteria (right dotted line), where 5 nonzero coefficients were selected. LASSO, least absolute shrinkage and selection operator; SE, standard error

Among the study participants, 30.4% (213/700) were MDR-TB patients. Demographic and clinical characteristics of study participants are shown in Table 1. Of the 14 variables collected from patients, 5 variables were selected based on non-zero coefficients calculated by LASSO regression analysis (Fig. 1). These variables included completed secondary education, previous history of TB, any side effect of treatment, history of hospitalization, and spoligotype.

**Table 1:** Demographic and clinical characteristics of study participants

Characteristic	TB patient		P-value
	MDR-TB (n = 213)	DS-TB (n = 487)	
Gender, n (%)			0.962
Male	84 (39.4)	193 (39.6)	
Female	129 (60.6)	294 (60.4)	
Alcohol use (≥ one unit/day), n (%)			0.606
Yes	26 (12.2)	53 (10.9)	
No	187 (87.8)	434 (89.1)	
Smoking (any cigarettes/week), n (%)			0.256
Yes	38 (17.8)	70 (14.4)	
No	175 (82.2)	417 (85.6)	
Previous TB history, n (%)			<0.001
Yes	68 (31.9)	62 (12.9)	
No	145 (68.1)	425 (87.1)	
HIV positive, n (%)			0.020
Yes	18 (8.5)	20 (4.1)	
No	195 (91.5)	467 (95.9)	
Coexisting diabetes, n (%)			0.518
Yes	14 (6.6)	26 (5.3)	
No	199 (93.4)	461 (94.7)	
History of hospitalization, n (%)			0.257
Yes	14 (6.6)	22 (4.5)	
No	199 (93.4)	465 (95.5)	
Any side effects of treatment, n (%)			<0.001
Yes	145 (68.1)	206 (42.3)	
No	68 (31.9)	281 (57.7)	
Socioeconomic status^a^, n (%)			0.152
1	77 (36.2)	211 (43.3)	
2	73 (34.3)	137 (28.1)	
3	63 (29.6)	139 (28.5)	
Completed secondary education, n (%)			0.007
Yes	143 (67.1)	274 (56.9)	
No	70 (32.9)	213 (43.1)	
Employment status, n (%)			0.114
Unemployed	131 (61.5)	247 (50.7)	
Working	60 (28.2)	175 (35.9)	
Student	22 (10.3)	62 (12.7)	
Unknown	0 (0.0)	3 (0.1)	
Spoligotype family (SpolDB4 Database), n (%)			<0.001
Haarlem	23 (10.8)	120 (24.6)	
Beijing	19 (8.9)	53 (10.9)	
Latin American Mediterranean	42 (19.7)	50 (10.3)	
T	76 (35.7)	67 (13.8)	
Other Euro-American^b^	10 (4.7)	51 (10.5)	
Orphan/no family	17 (8.0)	58 (11.9)	
Unknown (no data)	26 (12.2)	88 (18.1)	
Sputum smear grade, n (%)			0.100
0	26 (12.2)	41 (8.4)	
1	52 (24.4)	145 (29.8)	
2	47 (22.1)	133 (27.3)	
3	82 (38.5)	152 (31.2)	
Unknown	6 (2.8)	16 (3.3)	
Mean cough duration, (weeks)	4.0 (2.2–8.0)	4.0 (2.0–8.0)	0.112

*TB* tuberculosis, *HIV* human immunodeficiency virus, *MDR*-*TB* multidrug-resistant tuberculosis, *DS*-*TB* drug-susceptible tuberculosis

^a^Divided into three levels based on the scoring system used in the Peruvian National Census

^b^ “Other Euro-American” includes strains from the S family, the X family, and strains that were present in the SpolDB4 Database but had not yet been assigned a family [9]

**Fig. 2:**
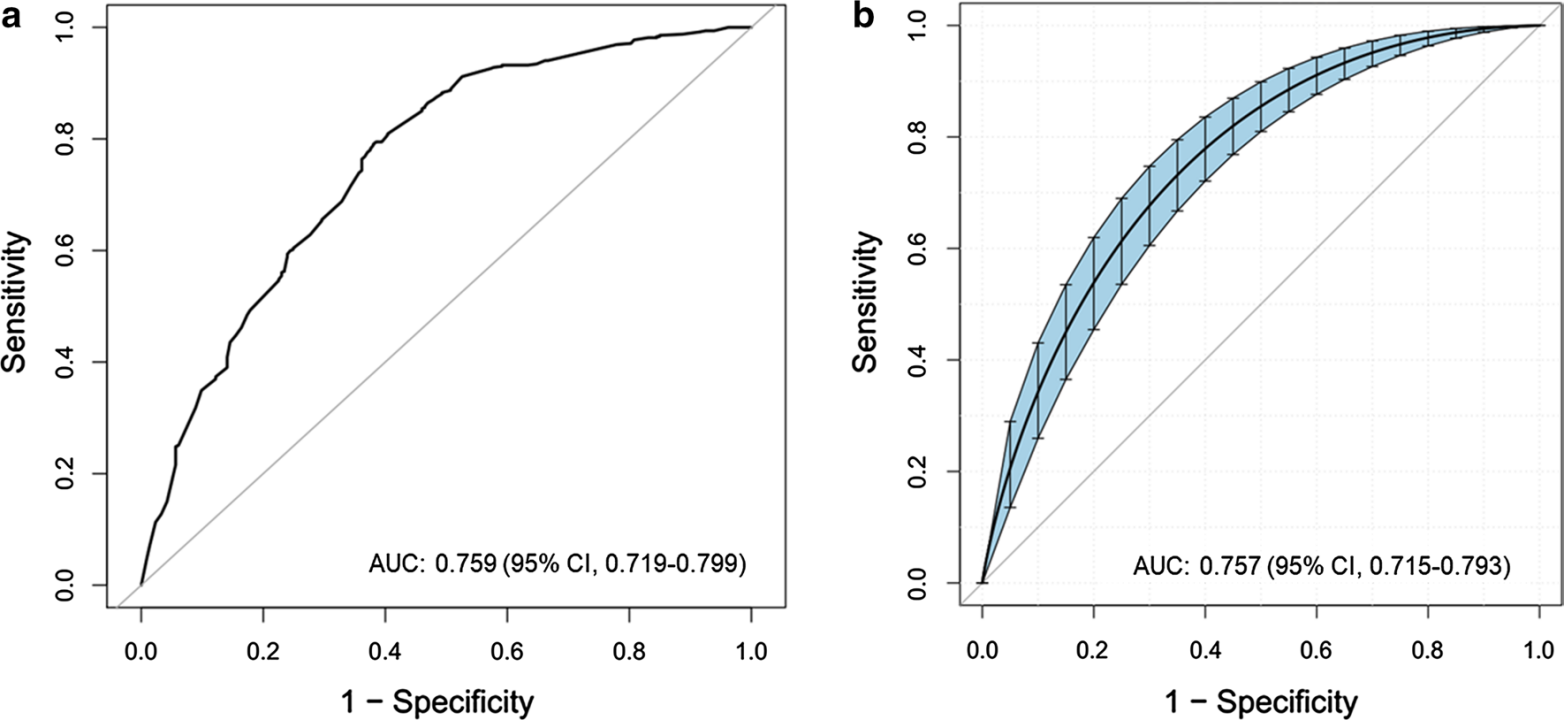
The AUC (representative the discriminatory ability of the model) of the model and the internal validation. (A) shows the AUC of the predictive model, and (B) shows the AUC of the internal validation using the bootstrap method (resampling = 500). The dotted vertical lines represent the 95% confidence interval. AUC, area under the curve

**Fig. 3:**
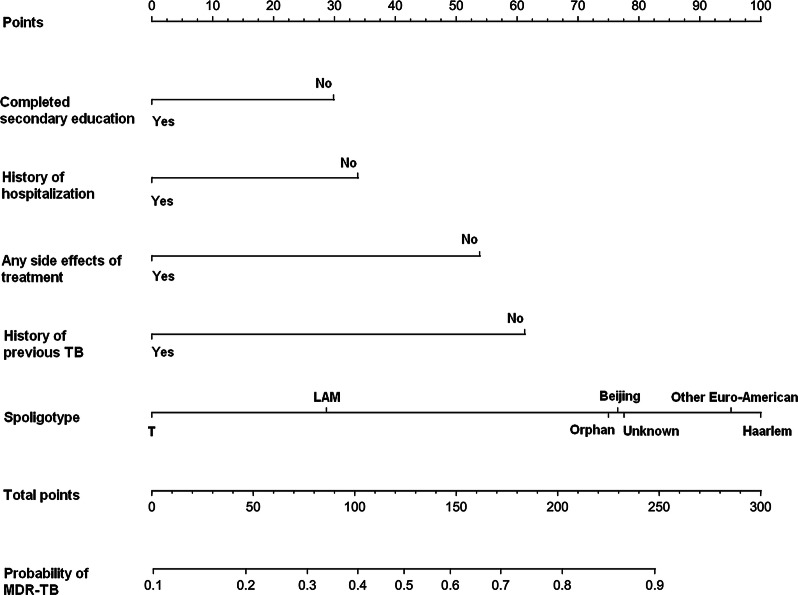
Nomogram for predicting MDR-TB risk and its algorithm. First, a point was found for each variable of a TB patient on the uppermost rule; then all scores were added together and the total number of points were collected. Finally, the corresponding predicted probability of MDR-TB was found on the lowest rule. “Other Euro-American” includes strains from the S family, the X family, and strains that were present in the SpolDB4 Database but had not yet been assigned a family [9]. MDR-TB, multidrug-resistant tuberculosis

To develop a predictive model for MDR-TB, multivariable logistic regression analysis was performed based on the aforementioned 5 variables selected by the LASSO regression technique. The AUC for the predictive model was 0.759 (95% confidence interval [CI], 0.719–0.799), and the internal validation using the bootstrap method (resampling = 500) was 0.757 (95% CI 0.715–0.793) (Fig. 2). To present the predictive model, a nomogram was constructed, thereby providing a convenient, personalized tool to predict the probability of MDR-TB (Fig. 3).

**Fig. 4:**
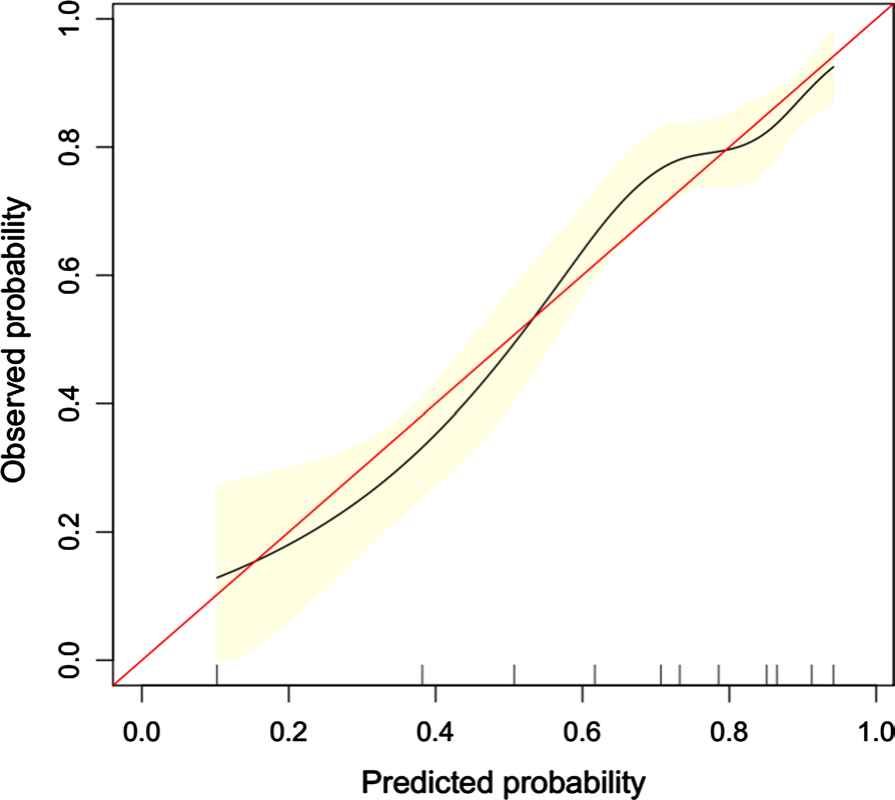
Calibration curve of the predictive model showing the degree of consistency between the predicted probability and observed probability (the Hosmer–Lemeshow test, P > 0.05, suggesting that it is of goodness-of-fit). The red solid line represents a perfect prediction by an ideal model, and the solid black line shows the performance of the model. The yellow shadow represents the 95% confidence interval

The proposed model was well-calibrated (Fig. 4). The Hosmer–Lemeshow test yielded a nonsignificant P value of 0.452, thereby suggesting that there was no statistical departure from a perfect fit between the predicted and observed values.

**Fig. 5:**
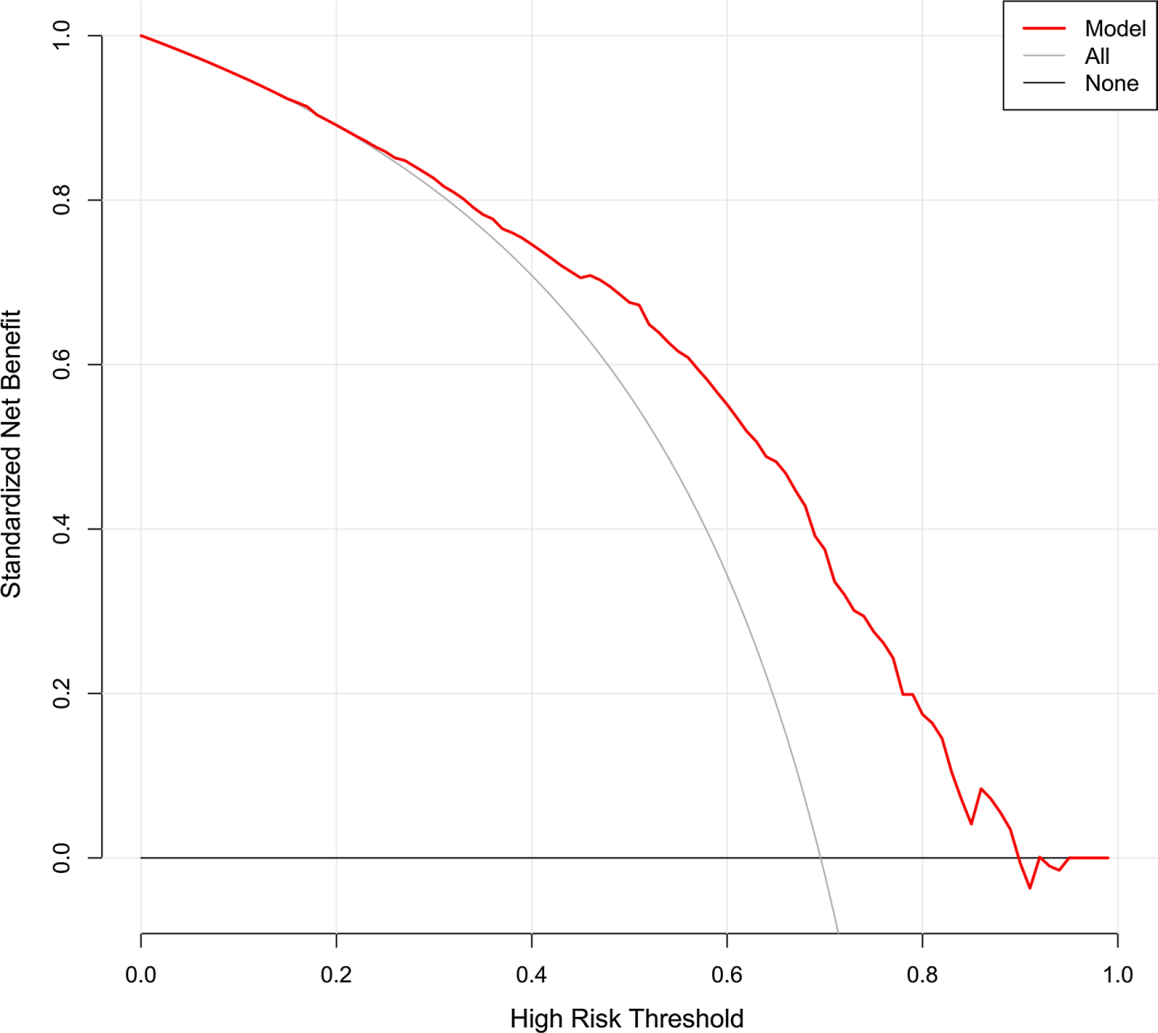
DCA of the nomogram. The red solid line represents the nomogram. The decision curve indicates that when the threshold probability of MDR-TB is between 70% and 90%, application of this nomogram would add a net benefit when compared with either the treat-all or the treat-none strategies. DCA, Decision curve analysis; MDR-TB, multidrug-resistant tuberculosis

To assess its clinical usefulness, DCA was also performed. The decision curve showed that when based on the nomogram in this study, the threshold probability of MDR-TB in TB patients was of 70–90% (Fig. 5), and application of this nomogram to predict MDR-TB would add significantly more benefit than either the treat-all scheme or the treat-none scheme.

## Discussion

In the present study, a nomogram for predicting MDR-TB among TB patients was built. This nomogram incorporated 5 variables, including completed secondary education, previous history of TB, any side effect of treatment, history of hospitalization, and spoligotype. The nomogram showed good discriminatory ability, calibration, and clinical usefulness.

DR-TB has always been a public health crisis and a health security threat [1]. Alarmingly, in the last few decades, the number of detected cases of MDR-TB dramatically increased in several countries, including India, China, Indonesia, the Philippines, Pakistan, Nigeria, Bangladesh, and South Africa [1]. Therefore, the possibility of unsuspected drug-resistance should always be considered when assessing a TB patient in any country or region in clinical practice. The WHO estimated that there were 558,000 new cases with resistance to rifampicin, of which approximately 82% had MDR-TB in 2017 [1]. Although DR-TB accounts for only 5% of TB, the costs involving the diagnosis and treatment of DR-TB are approximately 1/3 of that of global TB [1, 3]. Noteworthy, in endemic countries that utilizes traditional regimens for TB patients, those with DR-TB were associated with a staggering mortality, 40% in MDR-TB and 60–70% in extensively DR-TB (XDR-TB) [2]. At present, only 55% of MDR-TB patients are successfully treated worldwide [1].

Although MDR poses a challenge for treatment, in general, most MDR-TB patients can be cured by early detection of drug-resistance and using appropriately designed treatment regimens. MODS is a traditional method used to test rifampicin or isoniazid resistance in DST, and remains a major method used in some countries and regions. The main drawback of this method is that it is time-consuming and easy to miss the diagnosis. In recent years, the use of phenotypic DST has significantly expanded since the rapid test Xpert MTB/RIF was first recommended by the WHO in 2010 [1]. The advantage of this method is that it simultaneously detects TB and drug resistance to rifampicin in a very short time. Other molecular detection tests include array-based platforms and line probe assays [4, 5]. However, the aforementioned techniques for the identification of MDR-TB relies on sputum samples and are still unavailable in resource-limited settings [17]. Therefore, for MDR-TB patients with no sputum or without drug-resistance testing, or with sputum-negative TB, it is extremely easy to delay treatment or receive inappropriate treatment.

Regarding the risk of TB infection, several factors have been reported, such as smoking, poverty status, coexisting diabetes, HIV positive, and other conditions that lead to immunosuppression [1, 9]. In addition, previous studies have developed several models for predicting TB infection based on nosocomial populations, and it was pointed out by the authors that these models could improve the diagnosis of TB [18–20]. However, to our knowledge, few studies have described MDR-TB risk prediction models. In the current study, a nomogram for predicting MDR-TB was built based on 5 variables of TB patients. The variables included in the nomogram were filtered by LASSO regression analysis, which is considered superior to select predictors by univariate analysis [21, 22]. Furthermore, we evaluated the clinical significance of these predictors. Regarding “Education level of the patient”, it has been reported that economic status (poverty status) was associated with TB incidence [1, 9]. In general, the economic status is related to completion of education, and the economic level of those who completed education is higher compared to those who did not completed education. Therefore, we speculate that the “Education level” may associate with MDR-TB infection. Regarding the “History of hospital admission” and “side effects to drug”, it has been reported that poor adherence increases MDR-TB incidence [11], patients with a history of hospital admission may be better at treating adherence than patients who have never been hospitalized. Chaotic treatment (improper treatment programs) for TB has also resulted in an increase in MDR-TB incidence [11]. TB patients who received more chaotic treatment may experience more side effects to the drug. Hence, “History of hospital admission” and “side effects to drug” may be related to a risk of MDR-TB infection. These 5 predictors are easily available clinically. The nomogram showed good discriminatory ability and calibration, and the DCA evaluation showed its clinical usefulness. Since phenotypic DST has not yet been fully covered in TB-endemic areas, and many TB patients are sputum-free or sputum-negative TB, this cost-free nomogram may be helpful in screening MDR-TB in these TB patients.

This predictive model has several limitations. Firstly, this nomogram was built based on a 3-year prospective study conducted in Peru [9]. There is a regional difference in the prevalence of MDR-TB [1]; therefore, whether the nomogram is applicable to other regions or countries requires further multicenter verification. Secondly, it cannot be determined whether MDR-TB was acquired using this nomogram. Thirdly, some patient’s variables, such as regimens and duration of treatment were not included in our analysis because they were not available in the original dataset. Despite these limitations, this study was the first to develop a nomogram for predicting MDR-TB risk in TB patients.

## Conclusions

In this study, a nomogram for MDR-TB risk prediction was built, which incorporated five demographic and clinical characteristics of TB patients. This may be a useful complement to the current identification of MDR-TB, especially in patients with MDR-TB from whom test samples are not available.

## Data Availability

The data used in this study can be downloaded from ‘DATADRYAD’ database (http://www.Datadryad.org).

## Abbreviations

TB: Tuberculosis
LASSO: Least absolute shrinkage and selection operator
AUC: Area under the curve
DCA: Decision curve analysis
HIV: Human immunodeficiency virus
MDR-TB: Multidrug-resistant tuberculosis
